# Supplementary material for: Enhancing Physical Activity and Psychological Well-Being in College Students during COVID-19 through WeActive and WeMindful Interventions
Source: Int J Environ Res Public Health. 2022 Mar 31;19(7):4144. doi: 10.3390/ijerph19074144 (PMC8998224; doi:10.3390/ijerph19074144)
Supplement: Supplementary file 1 [file ijerph-19-04144-s001.zip › ijerph-1622784-supplementary.pdf]

Supplementary File

WeActive and WeMindful Lesson Plans.

**WeActive**	**WeMindful**
Intro/warmup<br>● 6 min<br>○ Marching in place<br>○ Single leg pull in<br>○ Marching plus arms circles<br>○ Sides steps<br>○ Side steps and overhead arm swings<br>○ Squat and hamstring roll -up<br>○ Lunge and twist<br>○ Overhead reach and side bend<br>Demo<br>● 1 min<br>Content<br>● 40 sec on 20 sec rest<br>● Repeat twice<br>○ Lower body circuit<br>■ Squats<br>■ Alternating lunges<br>■ High knees<br>○ Core circuit<br>■ Plank<br>■ Bird dogs<br>■ Leg lifts<br>■ Mountain climbers<br>Modifications given<br><br>Cool down and closure<br>● Walk around for 30 sec to lower HR<br>● Stretch 30–45 sec each<br>Closure review	Intro/warmup<br>● 5–7 min<br>○ Stand position cue proprioceptive awareness<br>○ Inhale 4 sec, exhale 6 sec (6x)<br>○ Reach up, exhale fold (4x)<br>○ Shake head, bent knees<br>○ Goal setting<br>Content<br>(18–20 min)<br>● 6 poses<br>○ Downward dog<br>○ 3 legged dog<br>○ Low lunge<br>○ high/crescent lunge<br>○ Warrior 2<br>○ Peaceful warrior<br>● Demo/practice together<br>● Put into flow 2–3x with breath<br>● Modifications given<br><br>Cool down and closure<br>(5 min)<br>● Stretch<br>● Deep breaths
Intro/warmup<br>● 5min (40sec per movement)<br>○ Marching in place<br>○ Marching + arm hugs<br>○ Single leg pull in<br>○ Side steps<br>○ Side steps +overhead arm swings<br>○ Forward bend + hamstring roll-up<br>○ Lunge + arm to ground twist<br>Demo<br>● 1 min<br>Content<br>● 40 sec on 20 sec rest<br>● Repeat 2–3<br>○ Lower body circuit<br>■ Wall sit<br>■ Side to side double hops<br>■ Stationary lunges	Intro/warmup<br>● 5–7 min<br>○ Inhale 4 sec, exhale 6 sec (6x)<br>○ Body scan<br>○ Goal setting<br>Content<br>(18–20 min)<br>● 3 new and 6 review poses<br>○ Downward dog<br>○ 3 legged dog<br>○ Low lunge<br>○ High/crescent lunge<br>○ Warrior 2<br>○ Peaceful warrior<br>○ Upward mountain pose/upward salute<br>○ Tree<br>○ Half standing forward bend

■ Butt kicks<br>○ Core circuit<br>■ Plank foot march<br>■ Parachutes<br>■ Flutter kicks<br>Modifications given<br><br>Cool down and closure<br>● Walk around for 30 sec to lower HR<br>● Stretch 30–45 sec each<br>Closure review	● Demo/practice together<br>● Put into flow 2–3x with breath<br>● Modifications given<br><br>Cool down and closure<br>(5 min)<br>● Stretch<br>● Deep breaths
Intro/warmup<br>● 5 min (40 sec per movement)<br>○ Marching + arm swings<br>○ Hip forward/back swings<br>○ Forward bend + hamstring roll up<br>○ Squat + hamstring roll up<br>○ Lunge + arm to ground twist<br>○ Shoulder standing bilateral external rotations<br>○ Scapular pushups<br>Demo<br>● 1 min<br>Content<br>● 40 sec on 20 sec rest<br>● Repeat 2–3<br>○ Lower body circuit<br>■ Tempo squat<br>■ Forward and back double hops<br>■ Stationary alternating side lunges<br>■ Jump squats<br>○ Core circuit<br>■ Plank hand march<br>■ Superman<br>■ Deadbug<br>Modifications given<br><br>Cool down and closure<br>● Walk around for 30 sec to lower HR<br>● Stretch 30–45 sec each<br>Closure review	Intro/warmup<br>● 5–7 min<br>○ Standing body scan<br>○ Goal setting<br><br>Content<br><br>(18–20 min)<br>● 3 review and 5 new poses<br>○ Mountain<br>○ Tree<br>○ Half bend<br>○ Chair<br>○ Plank<br>○ Low plank (chaturanga)<br>○ Baby cobra<br>○ Child's pose<br>● Demo/practice together<br>● Put into flow 2–3x with breath<br>● Modifications given<br><br>Cool down and closure<br>(5 min)<br>● Sitting with eyes close<br>● Meditation mindful script excerpts from https://mindfulnessexercises.com/teen-meditation-to-believe-in-yourself/
Intro/warmup<br>● 5 min (45 sec per movement)<br>○ Marching + arm circles<br>○ Hip forward/back swings<br>○ Hip side to side swings<br>○ Stationary side lunge stretch<br>○ Lunge +arm to ground twist<br>○ Shoulder standing bilateral external rotations<br>○ Scapular pushups	Intro/warmup<br>● 5–7 min<br>○ Standing or sitting (participants choice) body scan<br>○ Goal setting<br><br>Content<br>(18–20 min)<br>● Midterm review<br>○ Downward dog

Demo<br>• 1 min<br>Content<br>• 45 sec on 15 sec rest (standing circuit)<br>• 40 sec on 20 sec rest (laying)<br>• Repeat 2x<br>○ Standing circuit<br>■ Stationary lunge<br>■ Singe hops in place<br>■ Wall sit kicks<br>○ Laying circuit<br>■ Push ups<br>■ Full roll up<br>■ Double<br>■ Parachute hold<br>Modifications given<br><br>Cool down and closure<br>• Walk around for 30 sec to lower HR<br>• Stretch 30–45 sec each<br>Closure review	○ 3 legged dog<br>○ Low lunge<br>○ High crescent lunge<br>○ Warrior 2<br>○ Peaceful warrior<br>○ Mountain<br>○ Tree<br>○ Half bend<br>○ Chair<br>○ Plank<br>○ Low plank (chaturanga)<br>○ Baby cobra<br>○ Child's pose<br>• Demo/practice together<br>• Put into flow 2x with breath<br>• Modifications given<br><br>Cool down and closure<br>(2–3 min)<br>• Sitting with eyes close<br>• Mindful meditation script written by instructor
Intro/warmup<br>• 5 min (45 sec per movement)<br>○ Marching + arm circles<br>○ Hip forward/back swings<br>○ Stationary side lunge stretch<br>○ Lunge +arm to ground twist<br>○ Squat roll ups<br>○ Squat + air angels<br>○ Scapular pushups<br>Demo<br>• 1 min<br>Content<br>• 45 sec on 15 sec rest (standing circuit)<br>• 40 sec on 20 sec rest (laying)<br>• Repeat 2x<br>○ Standing circuit<br>■ Walking lunges<br>■ Squat jumps<br>■ Single hops side to side<br>■ Single leg deadlift<br>○ Laying circuit<br>■ Close grip pushups<br>■ Spiderman plank<br>■ Single leg bridge<br>■ Flutter kicks<br>Modifications given<br><br>Cool down and closure<br>• Walk around for 30 sec to lower HR<br>• Stretch 30–45 sec each<br>Closure review	Intro/warmup<br>• 5–7 min<br>○ Breath reach overhead<br>○ Forward fold<br>○ Standing side stretch<br>○ Goal setting<br><br>Content<br>(18–20 min)<br>• 6 poses<br>○ Triangle pose<br>○ Cat/cow<br>○ Thread needle<br>○ Head to knee forward bend<br>○ Sit and twist<br>○ Laying pigeon<br>• Demo/practice together<br>• Put into flow 3–4x with breath<br>• Modifications given<br><br>Cool down and closure<br>(2–3 min)<br>• Sitting with eyes close<br>• Mindful meditation script written by instructor
Intro/warmup	Intro/warmup

<table>
<tr>
<td>
3 min (45 sec per movement)
Marching + arm circles
Hip forward/back swings
Squat roll ups
Inchworm to downdog
Scapular push up
Demo
1 min
Content
45 sec on 15 sec rest
Repeat 2x (cardio)
Repeat 3x (core)
cardio circuit
High knees
Jumping jacks
Butt kicks
Mountain climbers
3 way lunges
core circuit
Plank hand shakes
Bicycles
Bent-knee V up
Hollow hold
Modifications given

Cool down and closure
Stretch 30–45 sec each
Closure review
</td>
<td>
5–7 min
Extremely slow walk around mat
Quicker
Regular pace
Awareness of body, senses and space cues
3 Deep breaths/goal setting
Content
(18–20 min)
6 review poses add 1
Triangle pose
Cat/cow
Thread needle
Head to knee forward bend
Sit and twist
Laying pigeon
Pelvic lift
Demo/practice together
Put into flow 2x with breath
Modifications given
Cool down and closure
(2–3 min)
Laying body scan: progressive muscle relaxation
Breath
Tense and relax
breath
</td>
</tr>
<tr>
<td>
Intro/warmup
3 min (45 sec per movement)
Marching + overhead reach and bend
Wide squat roll ups
Inchworm to down dog
Rocking lunge
Demo
1 min
Content
45 sec on 15 sec rest
Repeat 2x (both)
cardio circuit
Side shuffle
Jumping lunges
Inchworm burpees
Frog jump
Plank jack
core circuit
Plank knee tap
Parachute to superman hold
Toy soldiers
Full sit up
Side plank
Modifications given

Cool down and closure
</td>
<td>
Intro/warmup
5–7 min
Mindfulness
Pick something in the room. Describe it using 3 senses
Pick a part of your body that is touching the ground/chair. Describe the sensation in 3 words
3 things you know to be true right in this moment
Goal setting
Body
Rainbow stretch over left right front back
Stretch forward legs in front
Roll neck
Content
(18–20 min)
1 review and 4 new poses
Pelvic raise
Happy baby
Superman
Boat
Supine twist
Hold stretch for 10 sec and strength for 2 sets of 8 sec
</td>
</tr>
</table>

<table>
<tr>
<td>
Stretch 30–45 sec each
Closure review
</td>
<td>

Demo/practice together
Put into flow 3–4x with breath
Modifications given

Cool down and closure
(2–3 min)

Laying down 2 breaths
Meditation script by the meditation initiative read
Slowly start moving body again to sitting

</td>
</tr>
<tr>
<td>
Intro/warmup

3 min (45 sec per movement)

Marching + overhead reach and bend
Rocking side lunge
Scapular pushups
Rocking lunge

Demo
1 min
Content

45 sec on 15 sec rest
Repeat 2x (both)

cardio circuit

Jumping side lunges
Star jump
Burpees
Plyometric pushups
Plyometric donkey kicks

core circuit

Plank dual arm and leg raise
Plank up-downs
Candlestick leg lift
V-sit
Russian twists

Modifications given

Cool down and closure
Stretch 30–45 sec each
Closure review
</td>
<td>
Intro/warmup

5–7 min

WOOP

Wish
Outcome
Obstacle
Plan

Body

Touch 3 things/parts of your body with your feet, knee wrist, elbow, shoulder

Content
(18–20 min)

Final review: 2 flows

Flow one

Triangle
Cat/cow
Thread needle
Gate pose
Head to knee forward bend
Boat pose
Pelvic raise
Superman
Laying pigeon
Supine twist
Happy baby

Flow two

mountain
Tree
Chair
Half standing bend
Down dog
3 legged dog
Lunge
High lunge
Warrior 2
Peaceful warrior
Plank
Low plank
Baby cobra
Child's pose

Demo/practice together
Each flow once with breath

</td>
</tr>
</table>

	• Modifications given<br><br>Cool down and closure<br>(2–3 min)<br>• Laying body scan<br>• Meditative script written by instructor read
